# Unravelling tonicity: Causes of confusion and pathways to clarity^☆^

**DOI:** 10.1016/j.crphys.2025.100161

**Published:** 2025-08-07

**Authors:** Serena Y. Kuang, Xiaoqi Yang, Xiaonan Li

**Affiliations:** a Department of Foundational Medical Studies, Oakland University William Beaumont School of Medicine, Rochester, MI, USA; b Department of Infectious Disease, Icahn School of Medicine at Mount Sinai, New York, NY, USA; c Department of Children Health Care, Children's Hospital of Nanjing Medical University, Nanjing, China

**Keywords:** Osmosis system, Osmolarity, Osmotic concentration, Osmotic pressure gradient, Tonicity

## Abstract

Tonicity is the most confusing concept in teaching about osmosis in physiology, biology, and many clinical disciplines. A total of seven causes (four superficial and three deep) have led to this confusion but have never been thoroughly clarified. In this article, we systematically address and resolve these causes through logical reasoning, which not only thoroughly clarifies what tonicity is, but also leads to an understanding of its physical nature and properties. Several key concepts are introduced in order to resolve the causes of confusion and lay a new theoretical foundation for studying osmosis. This article not only advances the teaching and learning of tonicity and osmosis but also provides new insights into how osmosis across the cell membrane should be studied.

## Highlights

• Identifies four superficial and three deep causes of the confusion around tonicity.
• Clarifies the great confusion about tonicity in physiology education.
• Analyzes and addresses the physical nature and properties of tonicity.
• Introduces new concepts essential to understanding osmosis and tonicity.
• Fills key gaps in the literature on osmosis-related concepts.
• Opens a nee page in teaching and research on osmosis and tonicity.

## 1 Introduction

Tonicity is an osmosis-related concept most frequently taught in physiology. It also appears in textbooks of biology (Fowler et al., 2023; Russell et al., 2008), pharmacology (Barbour-Taylor et al., 2024), and many clinical disciplines (Lander et al., 2018; Mount et al., 2022; Stachowska-Pietka et al., 2015; Ved et al., 2016; Williams et al., 2019). However, there is great inconsistency in its definitions, so that what tonicity is remains unclear (Vujovic et al., 2018; Kuang et al., 2022a). This confusion causes difficulty in teaching and learning of both tonicity and osmosis. After reviewing the literature and many textbooks across disciplines, we selected 14 definitions and classified them into 5 categories (Kuang et al., 2022a). The definitions in the first three categories define tonicity as a property of a solution: a solution's osmolality or osmolarity (Category 1); the effective osmolarity, effective osmolality, or effective osmotic pressure of a solution (Category 2); or an ability or effect of a solution or a particle on cell volume (Category 3). Definitions or descriptions in Category 5 are other definitions that do not convey a clear meaning, such as “by measuring plasma osmolarity for a more accurate estimate of the true ‘effective’ tonicity of body fluids” (Basic Medical Key). Category 5 is not discussed in this article.

Different from Categories 1, 2, and 3, definitions in Category 4 consider tonicity to be relative or relational between two solutions. For example,

• Tonicity is “the relative difference in osmolarity in different compartments” (Gould, 2010, p.5).
• “A semi-quantitative descriptor of the concentration of one solution compared to another is ‘tonicity’” (Caon, 2008, p.94).
• “The term tonicity is used to describe the osmolality of a solution relative to plasma” (Barrett et al., 2019, p.32).
• “Whereas a solution's osmolarity is based solely on its total solute concentration, its tonicity is a function of the concentration of nonpermeating solutes outside a cell relative to the concentration inside the cell, and it determines the behavior of a cell placed in the solution” (Stanfield, 2013, p.112).
• “Tonicity is a measure of the osmotic pressure gradient between two solutions” (Study.com).

Throughout this article, we will reason out why considering tonicity to be relative or relational is in the right direction, however, among the five sample definitions above, only one is accurate. For the convenience of our conceptual analysis, we use “x-osmotic” to stand for the terms “hyper-osmotic/iso-osmotic/hypo-osmotic”, and “x-tonic” for “hypertonic/isotonic/hypotonic”.

The causes leading to the confusion surrounding the definition of tonicity are not straightforward, but complex. We identified a total of seven causes, with four being superficial and three more deeply rooted. In this article, we aim to resolve the confusion surrounding tonicity in three steps. We will first address and resolve the four superficial causes, then the three deep causes, and finally, we will illustrate the physical nature and three properties of tonicity. By so doing, the truth of tonicity and the multiple ways to express it will become clear and the understanding of osmosis will be deepened. This article is a significant expansion and improvement of our published abstracts (Kuang et al., 2020a, Kuang et al., 2022a, Kuang et al., 2022b, Kuang et al., 2022c, Kuang et al., 2023a, Kuang et al., 2023b). Several new concepts introduced in the article lay a new theoretical foundation to study osmosis, especially osmosis across the cell membrane.

## 2 Four superficial causes and resolutions

The four superficial causes of confusion about tonicity are the following: tonicity is considered a property of a solution, which is inappropriate; the term “tonic” contains a hidden ambiguity; the coexistence of (conventional) osmolarity and effective osmolarity leads to considerable confusion in understanding tonicity; and the membrane-dependency of the impermeant solute particles (SP) in a solution involved in osmosis is often overlooked or not explicitly addressed.

### 2.1 Tonicity is not a property of a solution

Although the definitions of tonicity are inconsistent and confusing, it is common that if the term “x-tonic” (i.e., hypertonic, isotonic, or hypotonic) is used, it reflects tonicity (Russell et al., 2008; Stanfield, 2013). For instance, Solution 1 (S_1_, such as an IV solution) is considered hypertonic to Solution 2 (S_2_, such as plasma), or conversely, S_2_ is hypotonic to S_1_. Clearly, the term “hypertonic” is comparative in nature, as it compares a specific property related to S_1_ with the same property related to S_2_. This is why tonicity cannot be defined as a property of a single solution but rather as a relationship between two solutions. When people erroneously think of tonicity as a property of a single solution, it sets a barrier to defining and understanding tonicity. This is one of the primary reasons for the confusion surrounding the definitions of tonicity.

From the macroscopic perspective, osmosis between two solutions separated by a semi-permeable membrane can be considered as a water-competing game, with the concentrations of impermeant SP in these solutions as the two players. For example, if the impermeant SP concentrations in S_1_ and S_2_ are 300 mOsm/L and 200 mOsm/L, respectively, water will move toward S_1_. In this “water-competing game,” S_1_ wins and gains water, while S_2_ loses.

It should be noted that the impermeant SP of S_1_ and S_2_ do not truly have the ability to “attract” water. Using the “water-competing game” analogy to describe osmosis between two solutions is merely a pedagogical convenience at the macroscopic level. The driving force underlying osmosis originates from molecular thermodynamic mechanisms, which involve interactions among water molecules, impermeant SP, and the membrane at a microscopic level (Kramer and Myers, 2013; Joos and Greeman, 1951). However, understanding this mechanism requires knowledge of physics and a substantial amount of explanation. At the macroscopic level, because water moves from the water compartment to the solution compartment, it appears as if the impermeant SP “attract” water.

An alternative macroscopic explanation of osmosis is that it is driven by the difference in water potential between the two compartments. In this view, water moves from the compartment with higher water potential (the water compartment) to the one with lower water potential (the solution compartment). Since the true microscopic molecular thermodynamic mechanism has not yet been widely disseminated in biomedical fields, the descriptions “impermeant SP attract water” or “water potential difference pushes water to the solution compartment” serve as macroscopic, pragmatic explanations that facilitate teaching. Both describe the same phenomenon, namely osmosis. They are two sides of the same thing: the unequal distribution of solute particles and water molecules across the membrane.

Since tonicity cannot be attributed to any single solution, who or what “owns” tonicity? To illustrate this, consider a candy-competing game between two siblings. Suppose the older brother, being stronger, gained 120 pieces, while the younger brother, being weaker, got 90 pieces. To convey the outcome of the game, we can describe it in several ways:

• The older brother is stronger than the younger brother, or the older brother won the game, or the younger brother lost the game (a descriptive approach).
• The older brother scored 30 more points than the younger one, or the younger one scored 30 fewer points than the older one (using the point difference to express the result).
• 120 : 90 (a ratio expression, which is the most informative).

Regardless of how we describe the outcome, the older one can only “own” 120 points, not “120 : 90”, and the younger one can only “own” 90 points, not “120 : 90”. The game itself, as a comparison of the two players’ scores, is the true owner of the result. Similarly, in the context of osmosis as a “water-competing game,” S_1_ and S_2_ “own” 300 mOsm/L and 200 mOsm/L, respectively, but the game itself, the competition, is the owner of the comparison, i.e., tonicity. Therefore, tonicity is not a property of any single solution but rather a property of the water-competing game (osmosis).

A difference between the candy-competing game and the water-competing game is that “120 : 90” is the outcome of the candy-competing game, while “300 : 200” predicts the outcome of the water-competing game. However, both analogies demonstrate that tonicity cannot be attributed solely to S_1_ or S_2_; it is inherently a property of the water-competing game itself. This is why tonicity is a relational term between two solutions separated by a membrane.

Just as the outcome of any game can be expressed in multiple ways, tonicity (which reflects a comparison) can also be described using various approaches:

• Descriptive expression: S_1_ is hypertonic to S_2_, or S_2_ is hypotonic to S_1_.
• Difference expression: 300–200 = 100 (mOsm/L) or 200–300 = −100 (mOsm/L), meaning that the osmotic strength of S_1_ is 100 mOsm/L stronger than S_2_ or the osmotic strength of S_2_ is 100 mOsm/L weaker than S_1_.
• Ratio expression: 300 : 200 (the most informative representation).

Tonicity can also be expressed based on the outcome of osmosis, which will become clear later when we address the physical nature of tonicity.

Based on the analysis presented above, the definitions of tonicity in Categories 1, 2, and 3 have all been refuted because they attribute tonicity to a single solution.

### 2.2 The term “tonic” should Be understood from a thermodynamic perspective

The word “tonic” comes from the Greek *tonikos*, meaning “relating to tension or tone”. Tension or tone often represents stored or potential energy in a system. However, what is meant by the “tone” related to osmosis has never been clearly defined.

The term “isotonic” was coined by the botanist Hugo de Vries in the nineteenth century (Kuang et al., 2022c; Hamburger, 1911). In his experiments, if S_X_ caused a specific degree of cell shrinkage and S_Y_ produced the same degree of shrinkage in the same cell, S_X_ and S_Y_ were deemed “isotonic solutions” because they exhibited equal “water-attracting force.” de Vries seems to have been unaware that when S_X_ is isotonic to S_Y_, both S_X_ and S_Y_ are hypertonic to the intracellular fluid (ICF) of the cell, or conversely, the ICF of the cell is hypotonic to both S_X_ and S_Y_. Again, we know that solutes in any solutions do not have the ability to attract water by themselves, but at the macroscopic level, we still need to use the “water-competing game” analogy as if impermeant SP “attract” water for the convenience of teaching as follows.

From the thermodynamic perspective, this so-called “water-attracting force” is exactly the difference between the impermeant SP concentrations (transmembrane osmotic gradient). This force can be considered a physical “tone” that stores energy and can drive osmosis. Taking S_1_ and S_2_ as an example, the tone corresponds to 100 mOsm/L (300 - 200). Therefore, this “water-attracting force” or “tone” does not originate solely from S_1_ or S_2_ individually but is instead jointly created by both solutions. When it is said that S_1_ is hypertonic to S_2_, it means that S_1_ is at the higher concentration end of the osmotic gradient, while S_2_ is at the lower concentration end. A transmembrane osmotic gradient is the osmotic tone in the system where osmosis occurs. If S_1_ has 200 mOsm/L and S_2_ has 300 mOsm/L, this osmotic tone = 200–300 = −100 (mOsm/L), meaning the osmotic tone or force has the same magnitude but opposite direction in contrast to the first example.

For the convenience of teaching in biomedical fields, an alternative way to interpret “tone” is as follows: The impermeant SP concentrations in two solutions (e.g., 300 mOsm/L and 200 mOsm/L) involved in osmosis can be considered two “tones” contributing to the osmotic gradient, with tonicity being the comparison between these two so-called “tones”.

It is essential to clarify that the thermodynamic interpretation of the single “tone” (transmembrane osmotic gradient) is the accurate one, while the dual-tone interpretation is merely a pedagogical approach for ease of teaching because it is the difference between 300 mOsm/L and 200 mOsm/L that stores energy related to osmosis, not any single impermeant SP concentration.

### 2.3 The Co-existence of conventional osmolarity and effective osmolarity causes unnecessary confusion

The conventional conception of osmolarity, no matter whether it is defined as the total concentration of solute particles in a solution (Gould, 2010; Stanfield, 2013; Kibble and Halsey, 2009), or “the osmolar concentration expressed as osmoles per liter of solution” (Hall and Hall, 2021a, p54), or “Concentration of osmotically active particles, expressed as osmoles per liter or milliosmoles per liter” (Costanzo, 2022a, p12), applies to an ideal membrane that is only permeable to water. Since only impermeant SP contribute to osmotic pressure, when facing a non-ideal membrane that is also permeable to some species of SP, these definitions no longer work consistently because what does the term “osmoles” measure is unclear (i.e., whether it measures the impermeant faction of SP or both impermeant and permeant SP is not addressed). Therefore, the terms “effective osmolarity” (Hudson et al., 2017; Johnson, 2008; Kibble, 2020a) or “effective osmolality” (Rasouli, 2016) have emerged to refer to the concentration of the impermeant SP. The co-existence of two types of osmolarity causes several issues.

First, it causes inconsistency in the definitions of tonicity in Category 4. The first three tonicity definitions are (m-independent) osmolarity-based, whereas the last two are m-dependent impermeant SP-based because either “non-penetrating solutes” or “osmotic pressure gradient between two solutions” are exclusive to the permeant SP.

Second, the dual concepts of osmolarity and effective osmolarity lead to the usage of x-osmotic (hyper-osmotic/iso-osmotic/hypo-osmotic) and x-tonic (hypertonic/isotonic/hypotonic). Some literature distinguishes them as follows (Costanzo, 2022a; Doemling, 1968; Hall and Hall, 2021b): x-osmotic is used to compare the (m-independent) osmolarity of two solutions, while x-tonic is used to compare the (m-dependent) effective osmolarity of two solutions. However, when introducing the six types of body fluid disturbances, x-osmotic is frequently used (e.g., hyper-osmotic volume contraction or expansion, iso-osmotic volume contraction or expansion, and hypo-osmotic volume contraction or expansion (Costanzo, 2022b; Kibble, 2020b; Tobias et al., 2022)). In fact, osmosis occurs across the cell membranes in hyper- or hypo-osmotic volume disturbances due to the imbalance of the “effective osmolarity” in the extracellular fluid (ECF) and intracellular fluid (ICF). In this context, using x-osmotic but not x-tonic is inaccurate.

Third, it is common to see the following statement or a similar one: an iso-osmotic solution is not always isotonic (Silverthorn, 2016). If the two types of osmolarity are continuously used, this statement is not incorrect, but the resulting disorder described below is considerable. Assuming S_a_ and S_b_ are separated by a membrane and S_b_'s conventional osmolarity = 300 mOsm/L and effective osmolarity is 270 mOsm/L, then,

• A hyper-osmotic S_a_ (conventional osmolarity = 310 mOsm/L) can be hypertonic (if its effective osmolarity = 300 mOsm/L), isotonic (if its effective osmolarity = 270 mOsm/L), or hypotonic (if its effective osmolarity = 250 mOsm/L) to S_b_.
• An iso-osmotic S_a_ (conventional osmolarity = 300 mOsm/L) can be isotonic (if its effective osmolarity = 270 mOsm/L), or hypotonic (if its effective osmolarity = 250 mOsm/L) to S_b_.
• A hypo-osmotic S_a_ (conventional osmolarity = 290 mOsm/L) can be hypertonic, isotonic, or hypotonic to S_b_, depending on the effective osmolarity of S_a_ resulting from the interaction of the S_a_ and the given membrane.

In brief, if a solution is x-osmotic to another solution, whether it is hypertonic, isotonic, or hypotonic to another solution is unpredictable, if the membrane separating the two solutions is not given or specified (i.e., the concentration of the effective osmolarity cannot be determined because this concentration is m-dependent). Hence, stating that an x-osmotic solution is not always x-tonic is meaningless and misleading. The great relational disorder between x-osmotic and x-tonic indicates that osmolarity has not been accurately understood and defined.

In brief, first, osmolarity should only have a single definition; there cannot be two. Moreover, it should be applicable to both ideal and non-ideal membranes, which will be explained later. Second, without addressing the m-dependency of the impermeant SP, tonicity cannot be defined and discussed at all.

### 2.4 We formally and systematically addressed the membrane-dependency of osmosis-related concepts

Osmosis-related concepts include osmoles, osmotically active particles, single osmolarity, osmotic pressure, osmotic pressure gradient (difference), and tonicity. Since only the impermeant SP contribute to osmotic pressure and the concentration of the impermeant SP is m-dependent, we reasoned out that all of these osmosis-related concepts are impermeant SP-related, thus m-dependent and inherently exclude the permeant SP (Kuang et al., 2020b). Addressing the m-dependency of the impermeant SP is one of the prerequisites to accurately defining osmolarity. So far, the four superficial causes have been resolved. In brief, we have resolved the ownership of tonicity as well as the thermodynamic “tone” x-tonic refers to and addressed why a single accurate definition of m-dependent osmolarity is needed.

## 3 Three deep causes and resolutions

The four superficial causes result from three deep causes: the absence of an understanding of the two fundamental osmosis systems, the lack of an accurate single definition of osmolarity, and a missing key concept to describe a solution (Kuang et al., 2020b).

### 3.1 Two fundamental osmosis systems: the missing prerequisites to studying osmosis

Two fundamental types of osmosis systems were previously introduced in our published abstract on tonicity (Kuang et al., 2020a): a simple osmosis system, in which a membrane (m) separates a pure water compartment (H_2_O) and a solution compartment (S), denoted as S-m-H_2_O, and a composite osmosis system, where the m separates two solutions, denoted as S_1_-m-S_2_. This can be deconstructed into two mirrored simple osmosis systems:

S_1_-m-S_2_ = S_1_-m-H_2_O + H_2_O-m-S_2_

Osmolarity and osmotic pressure must be defined in a simple S-m-H_2_O, not in any isolated solution. Tonicity must be defined in a composite S_1_-m-S_2_ (Kuang et al., 2020a). This point will become clear.

### 3.2 Defining the single osmolarity in a simple S-m-H_2_O

**Fig. 1:**
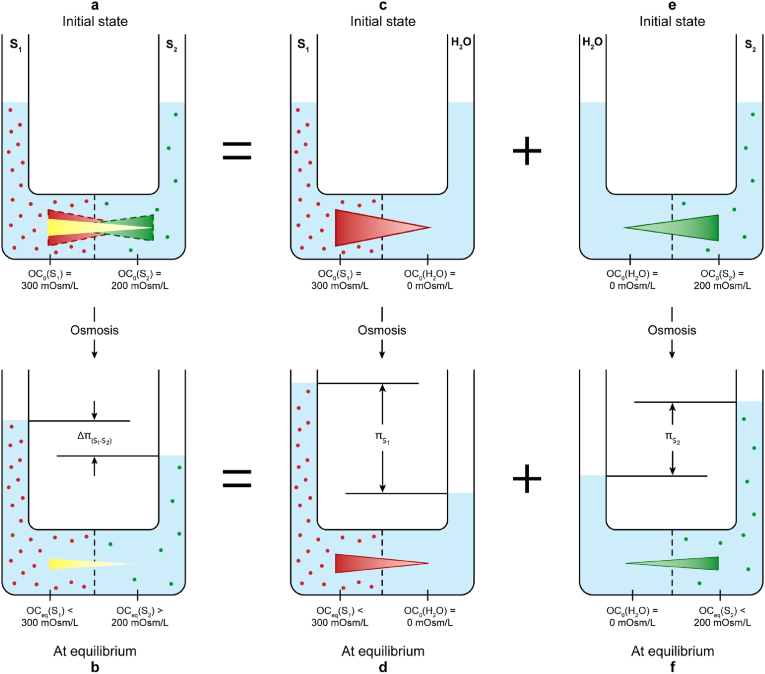
Deconstruction of the composite S_1_-m-S_2_ into two simple osmosis systems that are mirrored: S_1_-m-H_2_O and H_2_O-m-S_2_. a) The initial state of the S_1_-m-S_2_ before osmosis occurs. The yellow gradient across m indicates the difference between two OC_0_, where OC_0_ refers to the initial osmotic concentration (OC): ΔOC_0_(yellow) = OC_0_(S_1_) - OC_0_(S_2_). b) The equilibrium state of the S_1_-m-S_2_ after osmosis stops, where ΔOC_eq_ (yellow) = OC_eq_ (S_1_) – OC_eq_ (S_2_); OC_eq_ refers to the end OC at equilibrium (eq), S_1_ and S_2_ are the two solution compartments. c) The initial state of the simple S_1_-m-H_2_O with the red gradient showing the ΔOC_0_(red) = OC_0_(S_1_) - OC_0_(H_2_O) = OC_0_(S_1_). d) The end state of osmosis in the simple S_1_-m-H_2_O. e) The initial state of the simple H_2_O-m-S_2_ with the green gradient showing the ΔOC_0_(green) = OC_0_(H_2_O) - OC_0_(S_2_) = -OC_0_(S_2_) because OC_0_(H_2_O) = 0. The yellow gradient (in a) is the single thermodynamic “tone” in the composite S_1_-m-S_2_; without the deconstruction, it is simply calculated as OC_0_(S_1_) - OC_0_(S_2_); with the deconstruction, it becomes clear that the yellow gradient (in a) is a net gradient, i.e., the net gradient (yellow) = red gradient (in c) – green gradient (in e). The net gradient is a composite gradient that can be deconstructed into two simple gradients: the red and green gradients. π: osmotic pressure. Δπ: osmotic pressure gradient/difference. Image modified from Ref. (Kuang et al., 2020a), with permission from *The FASEB Journal*.

Osmolarity refers to the molar concentration of the impermeant SP of a solution in a simple osmosis system (not in an isolated solution (Kuang et al., 2020b),). Defined in this way, osmolarity is a parameter of the S-m-H_2_O system rather than of the solution in the S compartment because it is m-dependent: for the same solution, facing different membranes with varying permeability to the solute particle species in the solution, the resulting impermeant SP fraction is different. Osmolarity is osmotic concentration (OC). During osmosis, OC changes, so it is a variable. Its initial value, OC_0_ (i.e., before osmosis occurs or t = 0) is of practical use. The end value when osmosis stops (i.e., the system is at equilibrium (eq)) is OC_eq_ (see Fig. 1). When the membrane is ideal, 100 % of the total SP (TSP) in the S compartment are impermeant (Kuang et al., 2020b).

Osmosis across a cell's membrane takes place in a composite osmosis system, where S_1_ is ECF and S_2_ is ICF: ECF-m-ICF. 5 %glucose is considered isotonic to the plasma. After it is administered into the ECF, whether it is isotonic to the ICF depends on what cell membrane it faces. For cells impermeant to glucose (like some epithelial or connective tissue cells), it remains isotonic, but for glucose-permeable cells such as brain cells and red blood cells, which take up glucose via insulin-independent transporters (GLUT1, GLUT3), glucose quickly enters and is metabolized, lowering extracellular osmolarity, making the solution effectively hypotonic to the ICF of these cells. For insulin-dependent cells like muscle and fat, the effect depends on insulin levels: if insulin is present, glucose enters and the fluid becomes hypotonic; if not, it remains isotonic.

### 3.3 Introducing the new concept of **C_TSP_**: the source of OC_0_

**C_TSP_** (in boldface) is the molar concentration of total solute particles (TSP) measured in mM or M depending on context (1 M = 1000 mM), rather than in mOsm/L (Kuang et al., 2020b). **C_TSP_** is an inherent property of a solution, independent of whether the solution is part of an osmosis system or not, and its value is m-independent. **C_TSP_** is the source of OC_0_ when it interacts with a given membrane and differentiates into the permeant fraction (measured in mM) and impermeant fraction (i.e., OC_0_, measured in mOsm/L or Osm/L depending on context, 1 Osm/L = 1000 mOsm/L) in a simple S-m-H_2_O. Due to the absence of the concept of **C_TSP_** in describing a solution, conventional osmolarity has incorrectly taken its place and is instead measured in mOsm/L in physiology.

For example, the following equation appears frequently in physiology and many clinical textbooks to estimate the conventional osmolarity of plasma, with Na^+^ ions and glucose particles considered impermeant SP and urea a permeant species of SP:

P_osm_ = 2 x [P_Na_, mmol/L] + [P_glu_, mmol/L] + [P_urea_, mmol/L]

where P_osm_ = Plasma osmolarity (conventional osmolarity, mOsm/L); P_Na_ = Plasma Na^+^ concentration; P_glu_ = Plasma glucose concentration; P_urea_ = Plasma urea concentration, and [ ] represents concentration. In fact, what is calculated/estimated is the **C**_**TSP**_ of the plasma, which includes both the impermeant and permeant SP. In addition it is the **C**_**TSP**_ that determines the colligative properties of the solution, such as free point depression. When people apply the freezing point depression method to measure the osmolarity of a solution, what is measured is actually the **C**_**TSP**_ of the solution, not OC_0_ because the solution is not a part of any osmosis system.

To use a somewhat imperfect, non-academic analogy, **C**_**TSP**_ is like the “mother”, and the given membrane is like the “father”. When the two interact, they “give birth” to OC_0_, their “child.” When the “mother” is unrecognized, the child wrongly occupies the mother's position and is called (conventional) osmolarity. This kind of mismatch in the mother-child relationship is bound to lead to the emergence of another type of osmolarity (effective osmolarity) and cause confusion in the definition of tonicity. The analogy is imperfect because of the following mathematical relationship:

$${\text{OC}}_{0}=\frac{\left[m-\text{dependent}\;\text{impermeant}\;\text{SP}\right]}{C_{TSP}}$$

As shown in Table 1, the subtle yet crucial differences among conventional osmolarity, effective osmolarity, **C_TSP_**, OC, and OC₀ are highlighted. These subtle distinctions in Table 1 are keys to resolving the issues related to tonicity. Unit mM is used for **C_TSP_** and mOsm/L is for OC_0_ to be suitable for body fluids.

**Table 1:** Towards an accurate definition of osmotic concentration (OC).

	Definition and Ownership	Unit	Membrane-dependent	Constant or Variable	Effect or Role
**Conventional osmolarity**	Total solute particles (SP) of a solution	mOsm/L	No	Unclear, being used as a constant	A property of a solution; erroneously taking the place of **C_TSP_**
**Effective osmolarity**	Molar concentration of the impermeant SP of a solution	mOsm/L	Yes	Unclear, being used as a constant	A property of a solution, not a *simple* S-m-H_2_O
**C_TSP_**	Molar concentration of the total SP of a solution	mM	No	Constant	A property of a solution; used to replace conventional osmolarity
**OC**	Molar concentration of the impermeant SP of a *simple* S-m-H_2_O	mOsm/L	Yes	Variable (of no practical use)	Used to reason out OC_0_; a property of the system, not the solution
**OC_0_**	*Initial* OC before osmosis occurs	mOsm/L	Yes	Constant (of practical use)	A property of the system; used to replace effective osmolarity

It is the absence of the simple osmosis system, **C_TSP_**, and the m-dependency of osmosis-related concepts that leads to the co-existence of two types of osmolarity. It is the lack of a precise definition for the single osmolarity and an understanding of what is the “tone” x-tonic refers to that makes it difficult to understand the true meaning of “x-tonic”. It is the lack of the composite osmosis system that attributes tonicity to the wrong owner, that is, a solution.

### 3.4 Resolving the relational disorder between X-osmotic and X-tonic

In Fig. 1, the transmembrane red tone (ΔOC_0_(red)) pulls water to S_1_, whereas the transmembrane green tone (ΔOC_0_(green)) pulls water to S_2_. The transmembrane yellow tone [ΔOC_0_(yellow) = OC_0_(S_1_) - OC_0_(S_1_)] is the sum of the red and green tones, which pulls water to S_1_, resulting in the osmotic pressure difference (Δπ). Therefore, the “water-attracting force” described by de Vries in his experiment (Hamburger, 1911) did not unilaterally originate from S_X_ or S_Y_ but rather stems from the difference between OC_0_(S_X_) and OC_0_(ICF) or between OC_0_(S_Y_) and OC_0_(ICF). In this context, x-osmotic and x-tonic have the same meaning and can be used interchangeably. Hence, if a solution is hyper-osmotic/iso-osmotic/hypo-osmotic to another solution, it means it is hypertonic/isotonic/hypotonic to that solution. The unnecessary relational disorder between the terms x-osmotic and x-tonic is completely eliminated. The relationship between **C_TSP_** and OC_0_ is that OC_0_ is the m-dependent impermeant SP fraction of **C_TSP._**

Since both x-osmotic and x-tonic are comparative terms, the properties being compared must be homogeneous. We usually do not see statements such as “this solution is hyper-osmotic” and “that solution is hyper-osmotic to the cell”. However, we often encounter statements such as “this solution is hypertonic to the cell” or “that solution is hypertonic”. These statements are logically flawed. If a solution is only compared with the plasma and the truth of tonicity is crystal clear, “a solution is hypertonic to the plasma” may be simplified to “a solution is hypertonic.” Given the confusion that exists about tonicity, these statements make it even more difficult to understand.

The following statement, while not incorrect, fails to address the correct logic: If a solution causes a cell to shrink or swell, or if the cell volume at equilibrium has decreased or increased, the solution is hypertonic or hypotonic (Vujovic et al., 2018; Koeppen and Stanton, 2018; Sircar, 2014). It can be difficult to understand because it does not clearly explain why but requires students’ memory. The logic becomes clear if it is phrased differently: If a cell swells/shrinks in a solution, the cell won/lost the water-competing game (the winner gains water and volume and the loser lost water and volume).

By addressing the four superficial causes and three deep causes, all issues with tonicity are resolved as follows:

• **C_TSP_** interacting with a given membrane gives rise to the m-dependent OC_0_ in a simple S-m-H_2_O.
• The transmembrane thermodynamic tone in a simple S-m-H_2_O can be expressed as ΔOC_0_(simple) = OC_0_(S) – OC_0_(H_2_O) = OC_0_(S) because OC_0_(H_2_O) = 0, meaning that OC_0_(S) has no rival to compete for water during osmosis.
• The value and direction of ΔOC_0_(simple) in the simple S-m-H_2_O are absolute because they are unchangeable.
• ΔOC_0_(composite) in the composite S_1_-m-S_2_ is a net tone, which is distinct from the ΔOC_0_(simple) in the simple S-m-H_2_O (Fig. 1).
• ΔOC_0_(composite) is not absolute but relative, which is elaborated in the next section.

## 4 The physical nature and properties of tonicity

Tonicity has a two-sided physical nature and three properties.

### 4.1 Understanding the nature of tonicity in terms of energy transformation

As shown in Fig. 1a, during osmosis, the energy stored in ΔOC_0_(yellow) is transformed into the energy represented by Δπ. If applying a pressure that is equal to Δπ to reverse osmosis to its initial state, the energy stored in Δπ is transformed back into ΔOC_0_(yellow). Hence, the energy in the composite S_1_-m-S_2_ may take either the form of ΔOC_0_(yellow) or Δπ: During osmosis, the impermeant SP difference wanes and the hydrostatic pressure exerted by the increasing fluid column waxes; during reverse osmosis, the latter wanes and the former waxes. Therefore, the energy in the osmosis system is the unity of the impermeant SP difference and the hydrostatic pressure. ΔOC_0_(yellow) is the maximal impermeant SP difference when Δπ = 0, while Δπ is the maximal hydrostatic pressure when ΔOC_0_(yellow) reduces to ΔOC_eq_ (yellow). In other words, the ΔOC_0_(yellow) and Δπ are two complementary sides of the energy, where ΔOC_0_(yellow) represents the maximal active tone of the system as a driving force of osmosis (from the macroscopic perspective, not microscopic perspective), whereas Δπ represents the maximal, passive tone accumulating passively during osmosis (Kuang et al., 2023a). Both ΔOC_0_(yellow) and Δπ are m-dependent, therefore, they are both the system's parameters of the composite S_2_-m-S_1_, not any solution's.

Since “OC_0_(S_1_): OC_0_(S_2_)” predicts the result of the water-competing game, theoretically, calculating π_1_ and π_2_ using van ‘t Hoff's law and presenting them in the ratio format (π_1_: π_2_) can also predict tonicity, but in a less convenient way. Instead, the result itself, Δπ, can be a way to express tonicity. This is why tonicity can also be expressed using the result of osmosis and why the fifth sample definition in Category 4 (i.e., “Tonicity is a measure of the osmotic pressure gradient between two solutions” (Study.com)) is the only accurate one. The fourth sample definition in Category 4 is a bit convoluted: “tonicity is a function of the concentration of nonpermeating solutes outside a cell relative to the concentration inside the cell, and it determines the behavior of a cell placed in the solution” (Stanfield, 2013, p.112). If “a function of” is removed, it becomes clearer. Discussing tonicity solely based on whether a cell swells or shrinks is a narrow perspective that limits the application of tonicity. Tonicity can be applied to any composite S_1_-m-S_2_ systems: the given membrane does not have to be a cell membrane and one of the solutions in the system does not have to be the ICF of a cell.

In brief, tonicity has a dual physical nature, where ΔOC_0_(yellow, the ability) serves the predictive function and Δπ (the result) shows the outcome of the water-competing game directly.

### 4.2 Tonicity has three properties

The three properties of tonicity are illustrated below (Kuang et al., 2023a).

**Reciprocity:** Tonicity is reciprocal between two rival solutions and thus can only be addressed in terms of two solutions. Saying “S_1_ is hypertonic to S_2_” is equal to saying “S_2_ is hypotonic to S_1_”.

**Fig. 2:**
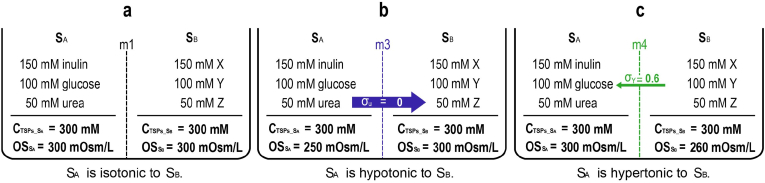
The membrane-dependency of tonicity. σ refers to a membrane's permeability to a particular species of solute particles (SP) (Costanzo, 2022a). If the membrane is impermeable to the SP (a), σ = 1; if fully permeable (b), σ = 0. σ between 0 and 1 (e.g., σ = 0.6 (c)) indicates partial permeability, meaning that the solute can cross the membrane to some extent, but not completely.

**Membrane (m)-dependency:** The m-dependency of tonicity is inherited from the m-dependency of OC_0_. Fig. 2 illustrates this property, where three different membranes separate the same two solutions, thus each scenario has its own m-specific tonicity.

Because tonicity is m-dependent, it only exists within a composite osmosis system that includes a specific membrane. Without a defined membrane, ΔOC_0_ cannot be generated, and tonicity does not exist, such as between two isolated solutions. Between two isolated solutions, only m-independent **C_TSP_** can be compared. Previously, we stated that the owner of tonicity is the water-competing game. Now, we can also say that the composite S_1_-m-S_2_ system is the owner of tonicity.

Returning to de Vries' experiment (Hamburger, 1911): Since both S_X_ and S_Y_ caused a cell to shrink by the same degree, he considered S_X_ to be isotonic to S_Y_. It should be noted that this isotonicity between S_X_ and S_Y_ only applies to this particular cell type. If he had used a different type of cell in the experiment and its membrane's permeability to the SP in the two solutions had been different, S_X_ and S_Y_ would not have been isotonic. For example, S_X_ might cause the second type of cell to swell, while S_2_ might cause it to shrink. This occurs because different types of cell membranes have different permeability properties for the same solutes in a solution.

**Relativity:** Tonicity is relative. If S_1_'s OC_0_ = 300 mOsm/L, S_2_'s OC_0_ = 200 mOsm/L, and S_3_'s OC_0_ = 350 mOsm/L, then S_1_ is hypertonic to S_2_ but hypotonic to S_3_. This means that tonicity is not only m-dependent but also reference solution-dependent: the size and direction of the transmembrane osmotic difference (the single thermodynamic tone) depends on the reference solution. Without specifying the reference solution (S_2_ or S_3_), the osmotic strength related to S_1_ cannot be determined and tonicity cannot be determined.

However, there is no tonicity in any simple osmosis system because of the lack of a reference solution, so the size and direction of the thermodynamic tone is absolute (never changes, i.e., any solution is hypertonic or hypo-osmotic to water). But in the composite S_1_-m-S_2_, first, the ΔOC_0_(yellow) is the net tone resulting from the sum of the red and green tones; and second, ΔOC_0_(yellow) is one way to express tonicity, while the red and green tones in two simple osmosis systems are not. Again, ΔOC_0_(composite) applies to S_1_-m-S_2_ and ΔOC_0_(simple) applies to S-m-H_2_O (see Fig. 1).

Table 2 summarizes and compares the system parameters in a simple S-m-H_2_O and a composite S_1_-m-S_2_, respectively.

**Table 2:** System parameters in two different osmosis systems. The units of all numbers are mOsm/L. van ‘t Hoff's law: π = RT⋅OC_0_, where R is gas constant and T is absolute temperature.

System	OC_0_	Active Tone	Passive Tone	Property
**S-m-H_2_O**	OC_0_(S)	ΔOC_0_(simple)	π = RT⋅OC_0_	Absolute, unchangeable
**Example**	300	300–0 = 300	π = 300RT	
**S_1_-m-S_2_**	OC_0_(S_1_) : OC_0_(S_2_)	ΔOC_0_(composite)	Δπ = π_1_ – π_2_	Relative, reference solution-dependent
**Example**	300 : 200	300–200 = 100	Δπ = 300RT – 200RT = 100RT	

### 4.3 An integrated view of tonicity in a composite S_1_-m-S_2_

**Fig. 3:**
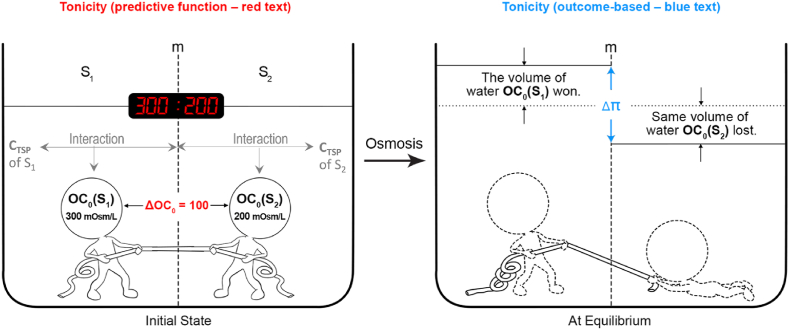
Osmosis in a composite osmosis system (S_1_-m-S_2_) represented as a tug of war, where the rope represents water. **C_TSP_:** molar concentration of the total particle solutes (TSP), the source of the initial osmotic concentration (OC_0_); m: a semi-permeable membrane that can be ideal or non-ideal; Δπ: osmotic pressure gradient or difference between S_1_ and S_2_, and Δπ = π(S_1_) – π(S_2_). Image modified from (Kuang et al., 2020a), with permission from *The FASEB Journal*.

Fig. 3 integrates **C_TSP_**, OC_0_, and tonicity in a composite S_1_-m-S_2_.

Understanding tonicity and the composite osmosis system is essential for studying osmosis in plant and animal physiology because the osmosis systems across cell membranes in living things are *composite*, not *simple*: ECF-m-ICF = ECF-m-H_2_O + H_2_O-m-ICF. It is clear now why tonicity is relational: A composite osmosis system contains two OC_0_, tonicity's role is to compare the osmotic strength between these two OC_0_.

## 5 Conclusions

The four superficial causes and three deep causes of confusion in teaching and learning about tonicity are resolved. Based on the logical analysis in this article, tonicity is a property of a composite osmosis system (not a simple osmosis system). It should be understood comprehensively in terms of its ownership, definition, multiple expressions, dual physical nature from the thermodynamic perspective, and three properties. The accurate definition of tonicity is as follows: tonicity reflects a comparison of the osmotic strengths in the two solutions (S_X_ and S_Y_) in a composite osmosis system (the owner of tonicity). The osmotic strengths of the two solutions can be expressed in multiple ways (Kuang et al., 2023b):

• S_X_ is x-osmotic or x-tonic to S_Y_ (no unit)
• OC_0_(S_X_) : OC_0_(S_Y_) (most informative and predictive; no unit)
• ΔOC_0_(composite) = OC_0_(S_X_) - OC_0_(S_Y_) (with a unit in mOsm/L)
• π_1_: π_2_ (inconvenient; no unit)
• Δπ = π_1_ - π_2_ (outcome-based; with a pressure unit such as mmHg or kPa (kilopascal)

There is a saying that tonicity has no unit, which is inaccurate. It depends on which form one wants to use to express tonicity. In addition, tonicity has a dual physical nature: either ΔOC_0_(composite) or Δπ. It also has three properties: reciprocal, m-dependent, and relative (reference solution-dependent).

In presenting these conclusions, this article opens a new chapter to advancing research and education on osmosis.

## CRediT authorship contribution statement

**Serena Y. Kuang:** Conceptualization, Formal analysis, Investigation, Methodology, Resources, Validation, Visualization, Project management, Writing – original draft, Writing – review & editing. **Xiaoqi Yang:** Conceptualization, Formal analysis, Investigation, Validation, Visualization, Writing – review & editing. **Xiaonan Li:** Conceptualization, Formal analysis, Investigation, Methodology, Resources, Validation, Visualization, Project management, Writing – review & editing.

## Declaration of competing interest

The authors declare that they have no conflicts of interest.

## Data Availability

No data was used for the research described in the article.
